# Temporal trend of the proportion of patients presenting with advanced HIV in French Guiana: stuck on the asymptote?

**DOI:** 10.1186/s13104-018-3944-y

**Published:** 2018-11-26

**Authors:** Mathieu Nacher, Florence Huber, Leila Adriouch, Félix Djossou, Antoine Adenis, Pierre Couppié

**Affiliations:** 1 Centre d’Investigation Clinique, CIC Inserm 1424, Cayenne Hospital, rue des Flamboyant, BP 6006, 97306 Cayenne Cedex, French Guiana; 2 COREVIH Guyane, Cayenne Hospital, rue des Flamboyant, BP 6006, 97306 Cayenne Cedex, French Guiana; 3 Service des Maladies Infectieuses et Tropicales, Cayenne Hospital, rue des Flamboyant, BP 6006, 97306 Cayenne Cedex, French Guiana; 4 Service de Dermatologie, Cayenne Hospital, rue des Flamboyant, BP 6006, 97306 Cayenne Cedex, French Guiana

**Keywords:** HIV, Advanced disease, CD4, AIDS, French Guiana

## Abstract

**Objective:**

In French Guiana, the French territory with the most preoccupying HIV epidemic, there have been great efforts to intensify and diversify HIV testing strategies. The aim of the present study was to review the temporal trends of patients diagnosed with advanced HIV disease in French Guiana. Data trends from the HIV cohort of French Guiana between 1996 and 2016 were thus analyzed.

**Results:**

The proportion of patients diagnosed with advanced disease did not decline over time. Males had lower CD4 counts at the time of diagnosis and there was a plateau for both males (around 40%) and females (around 25%) with no apparent reduction of the proportion of advanced disease. Older age groups and migrants presented more often with advanced disease. By contrast, the proportion of patients diagnosed with stage B and C disease declined over time and the CD4 count at antiretroviral initiation and the CD4 nadir increased over time. Despite some progress, the group of patients with advanced disease reached a plateau around 30% suggesting this particular group still has epidemiological importance in driving the epidemic and in fueling morbidity and mortality, and thus remains a challenge for testing strategies.

**Electronic supplementary material:**

The online version of this article (10.1186/s13104-018-3944-y) contains supplementary material, which is available to authorized users.

## Introduction

Since the first description of AIDS in 1981, the HIV epidemic has generated unprecedented progress in the development of effective drugs and therapeutic strategies. If every single patient was effectively treated, the ambition of a world without HIV would even be theoretically possible [1]. However, in real life, those who are infected and undiagnosed contribute disproportionally to the epidemic dynamics. A major goal is thus to continually reduce the proportion of undiagnosed HIV infections and to treat all HIV-infected patients in order to reduce morbidity and transmission.

French Guiana is the French territory with the most preoccupying HIV epidemic [2]. This French territory has access to all the newest antiretroviral drugs, PreP, residence permits and free universal health care for all HIV patients. About 75% of persons living with HIV are foreign nationals. The epidemic is driven by sex work, crack cocaine use, and multiple concurrent sexual partnerships [2]. The prevalence among pregnant women has been over 1% for 2 decades.

In French Guiana, males, older age groups and migrants were tested later on average than other groups [3]. In the past 5 years, there has been a great diversification of HIV testing strategies with ELISA lab testing, testing in emergency services, rapid testing at private practitioners, community based testing, mobile testing centers, and self testing [4]. The number of tests performed in French Guiana is also very high, more than twice of what is performed in Mainland France (205 tests per 1000 inhabitants versus 81 tests per 1000 inhabitants, respectively) [5].

Given the great efforts put into this strategic objective, the aim of the present study was to review the temporal trends of patients diagnosed with advanced HIV disease [6] in French Guiana.

## Main text

### Methods

Data on HIV patients in French Guiana has been available since 1989. Clinical, biological and epidemiological data was entered by specific trained research technicians in the DMI2 government software until 2008, and in eNADIS/DATAIDS since then, as described elsewhere [7, 8]. The data was aggregated by year. Given the small number of patients in the early years, this led to large fluctuations between years. Therefore, to avoid this “noise” we limited our analysis to the 1996–2016 period. This allowed us to obtain, for each year, the proportion of patients by CD4 count strata at the time of diagnosis, by CDC stage, by CD4 count strata at the time of treatment initiation, and the nadir CD4 count. This was stratified by sex, age group, and foreign/French status.

#### Statistical analysis

The main definition of “advanced disease” used was having CD4 counts < 200/mm^3^. To be more thorough we also looked at the CDC stage, the CD4 nadir, the CD4 count at the time of treatment initiation.

The data was plotted. In order to test for statistical trend in the curves we used regression models regressing the dependent variable on time, in practice testing the hypothesis that the slope in time was 0. The dependent variables were normalized when necessary to ensure homoscedasticity. Data was analyzed using STATA 13 software (College Station, Texas, USA).

#### Ethical and regulatory aspects

Patients included in the FHDH give written informed consent for the use of their data for research and publication of research results. Their identity is encrypted before sending the data to the Institut National de la Recherche Médicale (INSERM), which centralizes data from Regional Coordination for the fight against HIV (COREVIH) throughout France. This cohort has been approved since November 27th 1991 by the Commission Nationale Informatique et Libertés (CNIL) and has led to numerous international scientific publications.

### Results

Overall, between 1996 and 2016, there were 1965 patients analyzed (1053 females and 912 males). The transmission route was heterosexual contact for 86.8%, homosexual or bisexual contact for 3.7%, mother to child for 2.2% unknown for 6.6% and other transmission modes were < 1%.

**Fig. 1:**
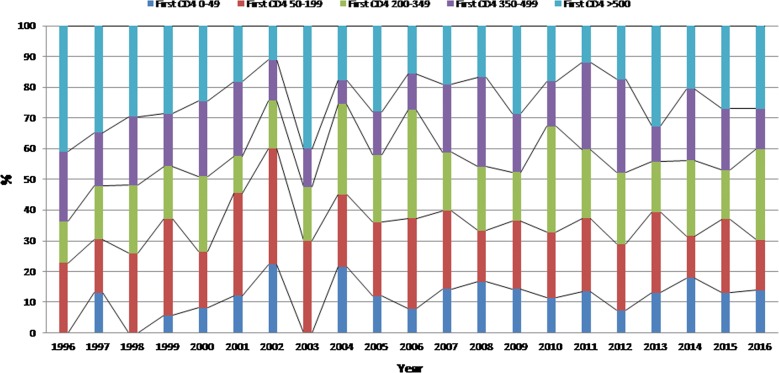
CD4 count at the time of diagnosis by year, French Guiana

**Fig. 2:**
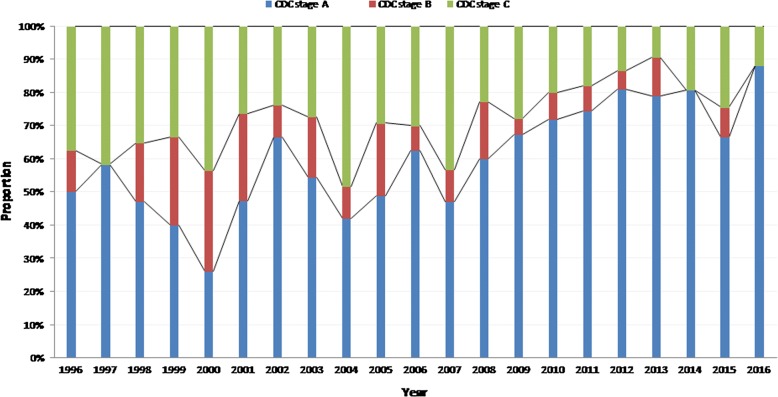
CDC stage when presenting to care in French Guiana: 1996–2016

**Fig. 3:**
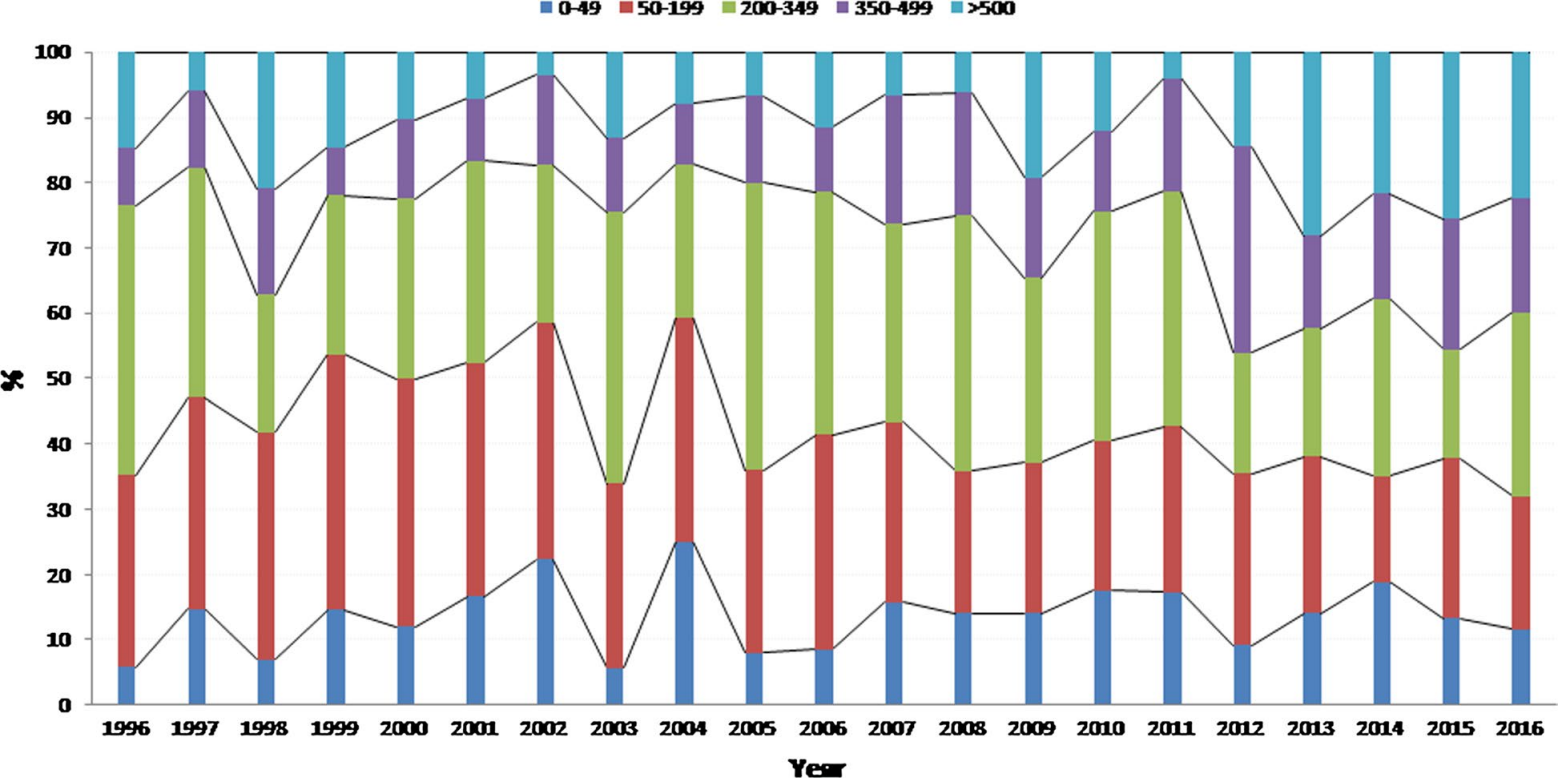
CD4 at initiation of antiretroviral therapy by year, French Guiana

Figure 1 shows that the proportion of patients diagnosed with advanced disease (CD4 > 200/mm^3^) did not decline over time. The linear regression model confirmed that there was no relation between time and the proportion of patients diagnosed with less than 200 CD4 lymphocytes per mm^3^ (Beta coefficient = 0.09, p = 0.75). Similarly those with less than 50 CD4 lymphocytes per mm^3^ did not vary with time (Beta coefficient = 0.2, p = 0.6). The apparent increase in the proportion of advanced disease until 2002 presumably reflects the recent history of the epidemic. Additional file 1: Figure S1 showed that males had lower CD4 counts at the time of diagnosis but that there was a plateau for both males (around 40%) and females (around 25%) with no apparent reduction of the proportion of those with CD4 count < 200/mm^3^ (Beta coefficient = 0.06, p = 0.88 and Beta coefficient = − 0.09, p = 0.8, respectively). Additional file 2: Figure S2 showed that older age groups presented more often with advanced disease. Given the stratification it was difficult to observe any significant trend. Additional file 3: Figure S3 showed that foreign patients were mostly involved in patients with advanced disease. The temporal trend analysis showed that foreign patients became more represented among patients with CD4 counts < 200/mm^3^ (Beta coefficient = 0.8, p = 0.03). Figure 2 showed that over time the proportion of patients first diagnosed with CDC stage B and C HIV-infection declined over time. This was confirmed by statistical analyses showing the trend was significant (Beta coefficient = − 1.27, p < 0.0001). Additional file 4: Figure S4 shows that the nadir CD4 count has gradually increased over time. This was confirmed by the linear regression model (Beta coefficient = 8.29, p < 0.0001). The proportion of patients with CD4 count at treatment initiation < 200 seemed to have slightly decreased (Beta coefficient = − 0.88, p = 0.003) but not those with CD4 counts < 50 at treatment initiation (Beta coefficient = 0.5, p = 0.8) (Fig. 3).

### Discussion

One of the objectives of the French Strategic National HIV/AIDS plan was to reduce by half the proportion of patients discovering their HIV diagnosis at the AIDS stage [9]. The new French National Strategy for Sexual Health now incremented HIV objectives from 90/90/90 to 95/95/95 but historical data suggests that diagnosing 95% of persons living with HIV may be very hard to achieve with the current testing paradigm [10].

The intensification of testing efforts, the multiplication of tools and strategies to enhance early testing, led to disappointing results in French Guiana. Although there has been a gradual decline in the proportion of patients diagnosed at stage C, this was not reduced by half as targeted. This objective thus seemed too optimistic. Males, older age groups and foreign nationals were most concerned with advanced disease. One hypothesis for the observed plateau of the proportion of advanced disease may be that given that the majority of patients are immigrants a significant proportion of them may have acquired HIV in their home country and arrive in French Guiana with an “old” HIV infection with significant immune suppression. In this scenario, if the patients were not yet in French Guiana in the early phases, testing them earlier is thus impossible. However, a recent study suggested that most immigrants acquired HIV within French Guiana [11, 12]. Interventions going towards the vulnerable communities supported by health mediators may lead to some progress in diagnosing persons who would not take the initiative to get tested for HIV. HIV-screening in association with other health priorities (hypertension, diabetes) may further increase HIV-testing uptake. Finally, studies in France have shown the frequent missed opportunities for HIV-testing of undiagnosed persons reporting frequent contacts with the health system before the diagnosis of HIV is actually performed [13]. Thus every contact with the health system should be an opportunity for testing. However, in practice this is often not done. Perhaps more systematic HIV testing when patients get routine blood examination, with a possibility to opt-out, may lead to a reduction of the proportion of patients with advanced disease [14, 15]. Much of the population already believes that any blood test goes with an automatic HIV test, which is a reason for erroneously thinking one is HIV-negative and does not need further testing. Therefore, opt-out testing would seem to be easy to implement from the point of view of the population because it would be to implement what many believe is already done. However, the present paradigm for health professionals and Non Government Organizations is opt-in testing and there is still some reticence to shift to a more utilitarian strategy.

Studies in Europe (COHERE, 33 cohorts from across Europe) showed some progress in reducing late presentation of HIV patients, but also showed that it remained a significant problem. In fact when looking at the proportion of patients with late presentation (< 350/mm^3^), with late presentation and advanced disease (CD4 < 200), and AIDS, it seemed that between 2006 and 2011 the decline reached a plateau, or was at best very modest [16]. This plateau aspect for advanced disease has also been observed in Sub Saharan Africa [17]. The proportion of patients with advanced disease at the time of diagnosis thus seems to converge to around 30% (in Greece it seems to have approached 20% [18]) of the total number of diagnosed patients, despite very different contexts. This sobering observation thus suggests that a significant proportion of persons live for years without knowing their diagnosis and thus continue to contribute disproportionally to the epidemic, and that opportunistic infections still have a bright future despite rapid therapeutic advances. Thus, in any population, there may be groups with different contextual elements, with different psychological attributes which lead to different attitudes towards health, prevention and testing. Some may have difficulties accessing care and some may have no motivation to do so. Whether the prevalence of certain psychological profiles or certain socioeconomic contexts stabilizes to 30% throughout the world remains to be determined. Reaching this group may be the most important challenge today if we are to have a “French Guiana without AIDS”. Indeed, in French Guiana we seem to be stuck around this asymptotic value of 30% of patients diagnosed with CD4 counts < 200/mm^3^.

However, French Guiana is in Latin America where a study in six countries showed there were 55% of late testers and 45% of late presenters (CD4 < 200), so 30% in that perspective is relatively “good” [19]. In addition, although the CD4 count at the time of diagnosis reached a plateau, the proportion of patients diagnosed because they had clinical signs (stages B and C) declined, therefore suggesting that improvements were made to test patients with clinically suspected immunosuppression. The challenge thus seemed to be those who were not clinically patent perhaps because they are less likely to come in contact with the health system.

Regarding CD4 count at antiretroviral initiation, between 30 and 40% on patients initiated ARVs with CD4 counts below 200 whereas in other parts of Latin America between 50 and 80% of patients have CD4 counts below 200 when starting ARVs [19]. This may have resulted from a combination of earlier diagnosis and broader antiretroviral treatment indications. The Nadir CD4 count showed a gradual increase, presumably resulting from a combination of scaling up of antiretroviral treatment and perhaps some overall progress in testing patients earlier.

In conclusion, despite efforts to intensify and diversify HIV testing, advanced disease remains a significant problem in French Guiana. Moreover, the proportion of patients with CD4 counts below 50/mm^3^ also remained constant. Thus, these patients who are long unaware of their diagnosis are presumably key in the propagation of the epidemic and also represent the group with the highest morbidity and mortality rates. In this context, opportunistic infections still have a bright future [7]. This observation challenges our current HIV testing strategies which fail to make progress in this group. Further studies should better describe circumstances and/or traits that underpin this observation.

## Limitations

These data show historical trends in French Guiana and some of the explanatory variables. However, these measures may be crude reflections of real life situation. In addition, the present results may not be easy to translate into optimal operational guidelines to reduce advanced disease. There is a definite lack of qualitative analysis of the stories behind each individual patient diagnosed with advanced HIV disease. These insights would be precious to better understand the trajectories, the missed opportunities, and potential avenues for reducing the frequency late diagnoses.

## Additional files

**Additional file 1: Figure S1.** Advanced HIV by sex French Guiana: 1996–2016.

**Additional file 2: Figure S2.** Advanced HIV by age group French Guiana: 1996–2016.

**Additional file 3: Figure S3.** Proportion of patients with advanced disease by foreign status.

**Additional file 4: Figure S4.** Nadir of CD4 count by year in French Guiana.

## Abbreviations

AIDS: acquired immuno deficiency syndrome
ARV: antiretroviral
CNIL: Commission Nationale Informatique et Libertés
INSERM: Institut National de la Santé et de la Recherche Médicale
HIV: human immunodeficiency virus
