# Supplementary figures and images for: Temporal trend of the proportion of patients presenting with advanced HIV in French Guiana: stuck on the asymptote?
Source: BMC Res Notes. 2018 Nov 26;11:831. doi: 10.1186/s13104-018-3944-y (PMC6258272; doi:10.1186/s13104-018-3944-y)

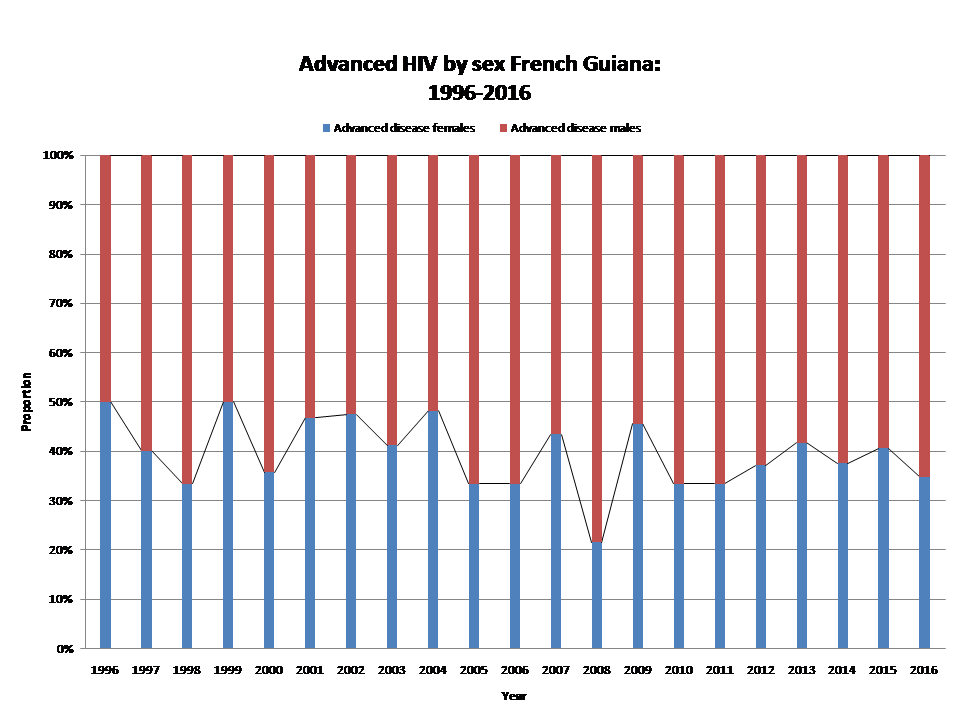

Supplement: Supplementary file 1 — Additional file 1: Figure S1. Advanced HIV by sex French Guiana: 1996–2016. [file 13104_2018_3944_MOESM1_ESM.tif]

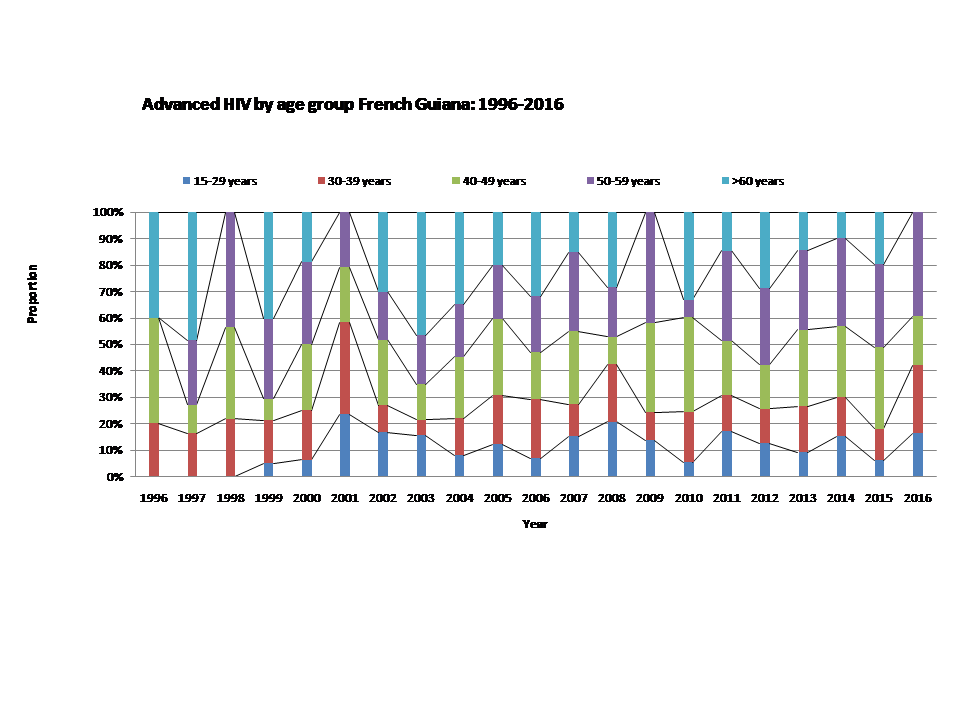

Supplement: Supplementary file 2 — Additional file 2: Figure S2. Advanced HIV by age group French Guiana: 1996–2016. [file 13104_2018_3944_MOESM2_ESM.tif]

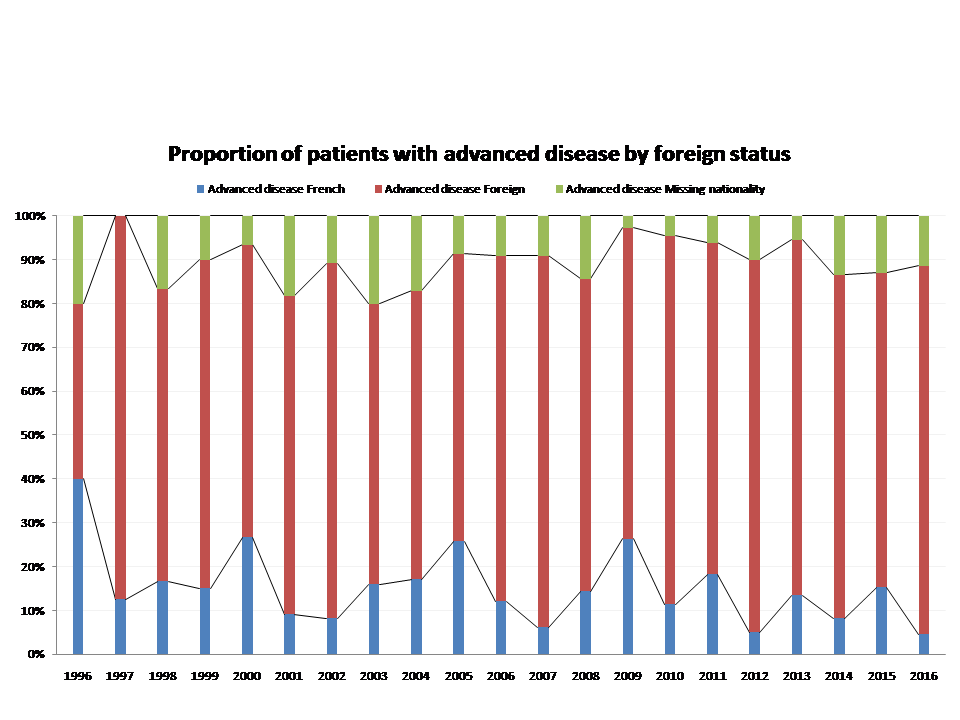

Supplement: Supplementary file 3 — Additional file 3: Figure S3. Proportion of patients with advanced disease by foreign status. [file 13104_2018_3944_MOESM3_ESM.tif]

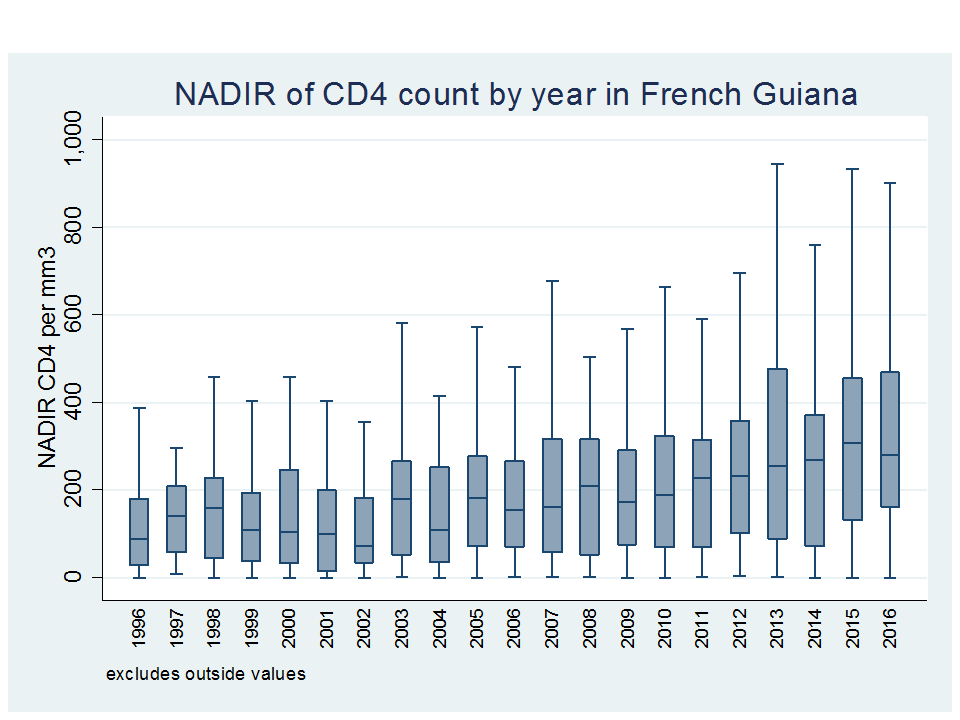

Supplement: Supplementary file 4 — Additional file 4: Figure S4. Nadir of CD4 count by year in French Guiana. [file 13104_2018_3944_MOESM4_ESM.tif]
